# Genomic fragment detection and infectivity evaluation of rotaviruses isolated from wastewater used for irrigation in western Bogotá, D. C.

**DOI:** 10.7705/biomedica.7935

**Published:** 2025-11-27

**Authors:** José Seir Jordán, Carlos Arturo Guerrero

**Affiliations:** 1 Laboratorio de Biología Molecular de Virus, Facultad de Medicina, Universidad Nacional de Colombia, Bogotá, D. C., Colombia Universidad Nacional de Colombia Universidad Nacional de Colombia Bogotá, D. C. Colombia

**Keywords:** Rotavirus, wastewater, environmental health, rotavirus, aguas residuales, salud ambiental

## Abstract

**Introduction.:**

Enteric viruses significantly impact morbidity, mortality, and healthcare. Transmission through wastewater is favoured in highly contaminated areas due to inadequate treatment.

**Objective.:**

To determine the number of rotaviruses and their infectious capacity from wastewater samples used for irrigation in the western part of Bogotá.

**Materials and methods.:**

Concentrations of group A rotavirus were monitored in wastewater using molecular methods. The infectivity of rotaviruses was evaluated in a mouse intestinal villi model. We assessed the feasibility of applying this approach for environmental health surveillance in Colombia, considering findings reported by other authors.

**Results.:**

The research focused on the *La Ramada* irrigation network in the western part of Bogotá, specifically the *Canal San José*. We analysed eighteen wastewater samples using qRT-PCR and detected group A rotavirus in twelve of them. The positive samples contained infectious rotavirus, as confirmed through the mouse villi model.

**Conclusion.:**

This study shows that contamination by group A rotavirus is frequent in wastewaters from the *Canal San José* in the *La Ramada* irrigation network in the western part of Bogotá and reveals high concentrations of rotavirus. The results suggest that villi from mouse intestines serve as a reliable model for isolating rotavirus from wastewaters. These findings provide a new approach for environmental health surveillance in Colombia, based on molecular epidemiology for waters highly contaminated with human enteric viruses.

## Resumen

**Introducción.:**

Los virus entéricos impactan significativamente la morbilidad, la mortalidad y los costos de atención médica. Su transmisión por medio de aguas residuales se potencia en zonas muy contaminadas debido al tratamiento deficiente de dichas aguas.

**Objetivo.:**

Determinar la cantidad de rotavirus y su capacidad infecciosa a partir de muestras de aguas residuales utilizadas para riego en el occidente de Bogotá.

**Materiales y métodos.:**

Se monitorearon concentraciones de rotavirus de tipo A en aguas residuales mediante métodos moleculares. La capacidad infecciosa de los rotavirus se evaluó en un modelo de vellosidades intestinales de ratón. Se analizó la posibilidad de utilizar este enfoque para la vigilancia de la salud ambiental en Colombia, tomando como referencia resultados similares reportados por otros autores.

**Resultados.:**

La investigación incluyó la red de riego La Ramada, al occidente de Bogotá, particularmente el *Canal San José*. Dieciocho muestras de aguas residuales fueron analizadas mediante qRT-PCR. Se detectaron rotavirus de tipo A en doce de ellas. Las doce muestras positivas contenían rotavirus infecciosos, como se comprobó en el modelo de vellosidades intestinales de ratón.

**Conclusión.:**

Los resultados muestran que la contaminación por rotavirus de tipo A es frecuente en aguas residuales del *Canal San José* de la red de riego *La Ramada*, al occidente de Bogotá, y revela grandes concentraciones del virus. Los resultados sugieren que las vellosidades del intestino de ratón sirven como modelo fiable para el aislamiento de rotavirus presentes en aguas residuales. Estos hallazgos proporcionan un nuevo enfoque de vigilancia de la salud ambiental en Colombia basado en epidemiología molecular para aguas muy contaminadas con virus entéricos humanos.

Several researchers have analyzed wastewater to track viruses from human feces ^1^^-^^6^. For example, one study found a SARS-CoV-2 concentration of 6.8 ×10^10^ copies per gram of human feces ^7^. Likewise, rotavirus concentrations between 20 to 7.2 × 10^2^ copies/ml have been reported in raw wastewaters ^5^^,^^8^^,^^9^. Enteric viruses are shed into sewage, subsequently contaminating rivers ^3^^,^^8^, and irrigation networks of raw vegetables ^10^. These facts suggest that wastewater -before, during, and after treatment- could be used to monitor human enteric viruses as a non-invasive early warning tool, assess infection trends, and guide public health responses ^1^^,^^2^^,^^11^^-^^14^.

It is important to consider the use of wastewater as an epidemiological surveillance indicator in Colombia. This approach would allow monitoring of water-related diseases by analyzing pathogens content in the water ^12^ without using invasive procedures (which are expensive for the healthcare system). Additionally, it would expand diagnostic coverage to the entire population from which the wastewater originates ^11^^,^^12^, enabling the identification of disease patterns in specific population groups, the projection and modeling of their epidemiological behavior ^1^, and the optimization of the already limited economic resources of the healthcare system in Colombia.

In 2016, fewer than 50% of the municipalities in Colombia had wastewater treatment plants ^15^, of which about 17% performed only primary treatment, accounting for nearly 43% of the total wastewater in the country ^16^. Therefore, additional data are needed to estimate the contribution of wastewater to the contamination of irrigation waters and, consequently, raw vegetables during their production ^10^^,^^17^. For example, the production area in the western part of Bogotá -called *La Ramada*-, located in the city’s flood zone, produces most of the vegetables consumed in Bogotá. However, its irrigation waters have shown high levels of contamination. Very high levels of *Escherichia coli* have been reported in untreated wastewaters, with an average of 1.3 × 10^6^ colony-forming units (CFU) per 100 ml, in contrast to other areas, such as the rural zones of the municipality of Cota (with minimal or no contaminated groundwater used for irrigation) or *Ciudad Bolívar* (average of 2.64 × 10^2^ CFU of *E. coli* per 100 ml of irrigation water from rural aqueducts) ^10^.

Part of the water used for irrigation in *La Ramada* network comes from pumping the Bogotá river, which is already contaminated upstream with wastewater from the municipalities bordering Bogotá to the north -Chía, Cajicá, Tocancipá, Gachancipá, Suesca, and Chocontá- where only secondary treatment is applied. It also receives inputs from the municipalities of Cota and Villapinzón, where treatment plants were constructed in 2020 ^10^. Another part of the water used in *La Ramada* comes from the contaminated Balsillas river, located in the northwest of Bogotá, and the municipalities of Mosquera (wastewater remained untreated until 2019) and Funza (water received secondary treatment the same year). This water is then discharged into the Gualí-Tres Esquinas marsh, from which it is pumped for irrigation in the network ^10^.

Previous environmental and sanitary conditions facilitate the transmission of foodborne, zoonotic, or waterborne diseases -caused by parasites, bacteria, and viruses- to consumers, farmers, or animals, increasing the burden of communicable diseases in the healthcare system. Viruses present in irrigation waters may remain infective for human and animal communities; they can be dispersed in the air, soil, and water bodies through irrigation activities ^10^. Therefore, it is necessary to evaluate the infective potential of these viruses under these environmental conditions.

The objective of this work was to characterize rotavirus in wastewaters from La Ramada irrigation network in western Bogotá, particularly in the Canal San José, and propose a new approach for environmental health surveillance of human enteric viruses found in wastewater in Colombia.

## Materials and methods

### Sampling zone

**Figure 1:**
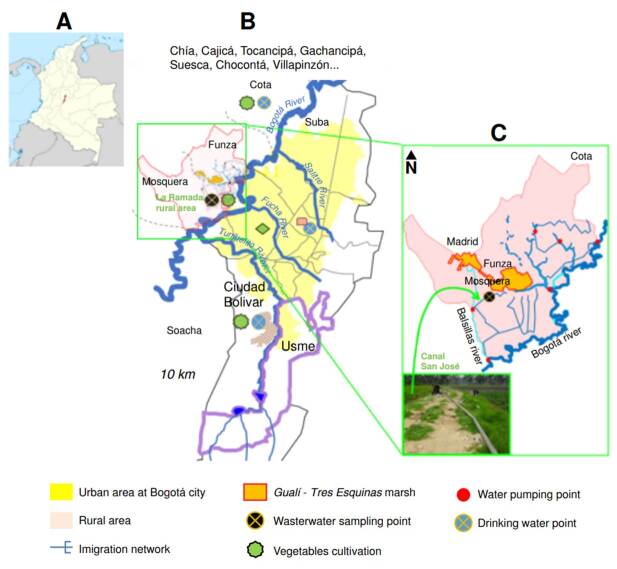
Geographiclocationofsamplingsites. A) Nationalscale(Colombia); B) Regionalscale (Bogotá); C) Local scale (*La Ramada*, rural area).

The study focused on the Andean region of Colombia (figure 1A), specifically *La Ramada*, a rural area in the western part of Bogotá (figure 1B), where an irrigation network is used for vegetable cultivation, including the so-called *Canal San José*. This canal is a runoff channel measuring approximately 1.5 m in width, 1 m in depth, and about 300 m in length (figure 1C). It collects wastewater from the Bogotá and Balsillas rivers and the Gualí-Tres Esquinas marsh through pumping stations. This water is used to irrigate various vegetable crops, including chard, lettuce, and spinach.

**Figure 2:**
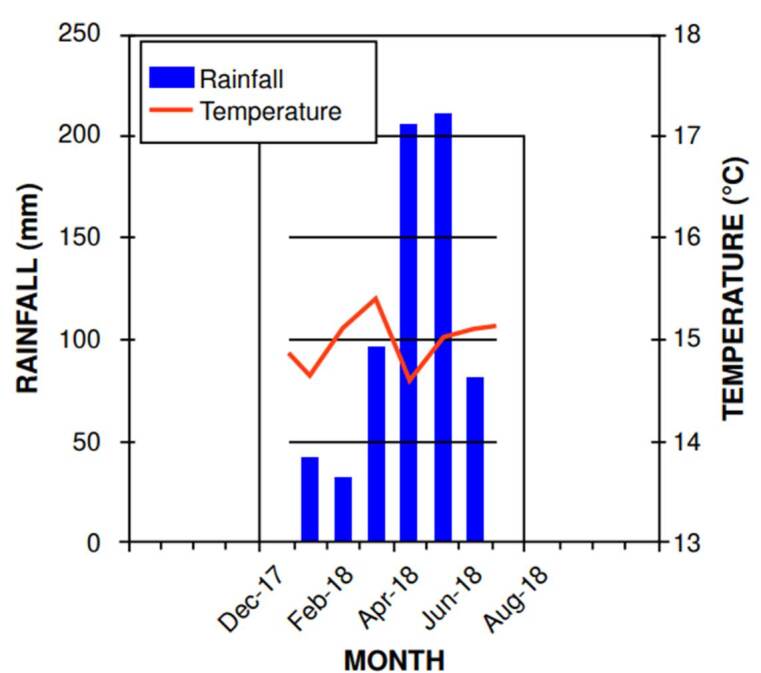
Monthly averages of temperature and rainfall during wastewater sampling at *Canal San José*, from June 2017 to November 2018, based on data from the Kennedy meteorological station (*Secretaría Distrital de Ambiente*)

This sampling zone was chosen due to its history as one of the largest production areas of vegetables sold in Bogotá. This city is located at 2,600 meters above sea level with a subtropical climate characterized by an average monthly temperature of 14-15 °C, an average annual rainfall of 1,013 mm, and precipitation of 100 mm or higher per month during April to May and October to November ^10^. During the sampling periods, annual precipitation ranged from 40 mm in low-rainfall months to over 200 mm in high- rainfall months (figure 2), likely due to climate change variability ^10^^,^^18^^,^^19^.

### Experimental design

Samples of wastewater were taken to monitor concentrations of group A rotavirus and evaluate their infectivity. The water was sampled monthly during low and high rainfall periods, between January and June 2018 (figure 2), at the *Canal San José* in *La Ramada* rural area. Water was collected at a depth of 20-30 cm and approximately 70 cm from the banks of the *Canal San José* using a 1.5 L low-density polyethylene beaker attached to a 1.8 m long stick. *In situ*, a 200 ml subsample was used to measure pH, temperature (°C), electrical conductivity (µS/m), and solids concentration (mg/L) using a multiparameter tester (HI98129, Hanna Instruments, Colombia). The time and sampling point were recorded for each measurement.

A second sample of 1 L was collected in a low density polyethylene flask to characterize the biological oxygen demand over five days (BOD_5_) and was stored at 0-4 °C in the *Laboratorio de Biología Molecular de Virus* of the *Universidad Nacional de Colombia* in Bogotá until its use after 24-72 h; a third sample consisted of 1 L of wastewater and was collected for quantitative reverse transcription polymerase chain reaction (qRT-PCR) testing or infectivity assessment, and was stored as previously described until its use in the experimental procedures. A total of eighteen wastewater samples were collected at the *Canal San José* for molecular testing, infectivity assessment, and physicochemical characterization; BOD_5_ analysis was performed on nine samples.

### Experimental procedures

The different collected samples were filtered and concentrated for subsequent rotavirus detection and, when applicable, infectivity assessment. Each concentrate was used for RNA extraction and infection assays. For the study, *rotavirus type* A was selected for detection, as it is the main cause of gastroenteritis in children under five years and the most common strain in humans. Therefore, primers and probes specific to group A rotavirus were used to evaluate this viral strain through qRT-PCR.

*Concentration and purification of rotaviruses in water*: To concentrate the viruses, each liter of the water sample was initially pre-filtered to remove large material using a 90 mm filter paper with 8-12 μm pores. Subsequently, the concentration process was performed using modified methods for water sample purification ^10^^,^^20^. Prior to the filtration of each 1,000 ml, the 90 mm hydrophilic mixed cellulose ester filter with 0.45 μm pores was treated with 5 ml of 250 mM AlCl_3_ to promote rotavirus adsorption.

Viruses were eluted from each filter using 200 ml of 0.5 mM H_2_SO_4_ at pH 3.0, and then, 10 ml of 1 mM NaOH at pH 10.8. To neutralize the pH of the eluate, 50 μl of 100 mM H_2_SO_4_ at pH 1.0 and 100 μl of 100X tris- EDTA at pH 8.0 were added per 10 ml of eluate. After this, solutions were supplemented with 1 g of polyethylene glycol 8000 (PEG 8000) per 10 ml of eluate. Mixtures were stirred gently at 4°C for 12 h and centrifuged at 4 °C and 10,000 rpm for 30 minutes.

The pellets were suspended in 2 ml of tris-base buffer at pH 8.0 (0.26 M Tris + 0.8 M NaCl). The 2 ml suspensions were concentrated in a Centriprep- YM-50 tube (Merck Millipore Sigma), reducing the suspension to 262-420 μl for subsequent rotavirus quantification.

*Quantification of rotavirus copies*: The viral RNA was extracted from concentrated and purified suspensions of viruses using the PureLink™ Viral RNA/DNA Mini Kit (Invitrogen, Thermo Fisher Scientific, Waltham, MA, USA), according to the manufacturer’s instructions; the internal controls and primers and probes for rotavirus type A amplification were acquired in the genesig^TM^ Advanced kits (Primerdesign^TM^ Ltd, Eastleigh, UK), with the non-structural protein 5 (NSP5) as gene target.

For qRT-PCR assays, we added 1 μl of primer and probe solution for the monitored virus, 1 μl of the primer and probe solution corresponding to the internal extraction control, 3 μl of water without DNase/RNase, and 10 μL of master mix solution (PrecisionPLUS OneStep RT-qPCR Master Mix (PrimerdesignTM Ltd, Eastleigh, UK) in a sterile 0.5 mL tube. We added 5 μl of the purified RNA extract to each tube.

For the standard curve, we prepared five serial ten-fold dilutions of the positive control. The 20 μl of each reaction mixture were dispensed into wells of a 96-well Hard-Shell® PCR HSP9645 plate (Bio-Rad, Hercules, CA, USA) and were covered immediately with a MicroAmp™ optical adhesive film. The plate was inserted into a CFX96 Touch™ Real-Time PCR detection system (BIO-RAD, Hercules, CA, USA), where we programmed the thermal profile according to the master mix manufacturer’s instructions. The qRT-PCR lasted 1 hour 55 minutes, and the obtained data were recorded and processed using the Bio-Rad CFX Maestro software, version 2.0, on Windows.

Copies of group A rotavirus were quantified using the standard curve. Each qRT-PCR result, expressed in terms of copies per five microliters of RNA, is the average value of three independent amplifications. This value was later converted into copies per liter of collected water sample (copies/L). Only results with quantification cycle (Cq) below 40 were considered positive. The detection threshold was calculated with the volumes of water sample and purified RNA extract added to the qRT-PCR mix. Water samples below the detection threshold were considered negative.

*Determination of rotavirus infectivity*: We use mouse intestinal villus methods to determine rotavirus levels of infectiousness in wastewater samples from the *Canal San José*^17^^,^^21^. Animal experiments were approved by the ethics committee of the Facultad de Medicina of the *Universidad Nacional de Colombia* in Bogotá, Colombia, according to the established guidelines (permission number 008-071- 16).

Briefly, adult male and female ICR mice (older than eight months), from the *Facultad de Medicina Veterinaria y de Zootecnia* at the *Universidad Nacional de Colombia*, were killed by cervical dislocation. The villi-enriched preparation in minimum essential medium (MEM) containing antibiotic/antimycotic solution was kept at 4 °C. The small intestine was flushed with MEM, cut into pieces, and treated with 1.5 mM EDTA and an antibiotic/antimycotic solution. Fragments were incubated at 37 °C with agitation, then dispersed by pipetting using a widened 1 ml tip. The suspension was filtered through a 1 mm^2^ metal net, and the retained material was resuspended for further dispersion and filtration. Both preparations were pooled, centrifuged at 600g for 5 minutes at 4 °C, and washed three times with MEM containing antibiotics. The final villus-enriched preparation was stored in MEM with antibiotics at 4 °C for later use in culture and virus infection.

*Rotavirus infection of isolated intestinal villi*: Intestinal villi isolated from mice were suspended in 4.5 ml of MEM and plated in 96-well cell culture plates (Nunc), ensuring equal distribution across wells. The villi were inoculated with 100 μl of concentrated samples pre-treated with trypsin (10 μg/ml) and incubated at 37 °C for 30 minutes. After 24 hours of culture at 37 °C, the villi were harvested. Rotavirus structural and non-structural proteins were detected by immunocytochemistry. The percentage of infected cells was estimated per triplicate and then converted into focus-forming units using the formula: UFF/ml = number of foci counted per inoculated volume (ml) per dilution; each sample was analyzed twice. Mock-infected villus cells served as a negative control, while ECwt rotavirus-infected cells acted as a positive control. The ECwt murine rotavirus strain was provided by doctor M. Franco (*Instituto de Genética*, *Pontificia Universidad Javeriana*, Bogotá, Colombia).

### Statistical analysis

Results are expressed as mean ± standard error (SE); n refers to the number of independent experiments performed for each analysis, generally in triplicate. We used a two-tailed Student t-test for comparisons and considered a value of p < 0.05 as statistically significant.

## Results

### Physicochemical characteristics of wastewater from the Canal San José

The physicochemical parameters of wastewater from the *San José Canal* -such as total dissolved solids, conductivity, and BOD_5_- evidenced significant contamination. Measurements taken during months of low and high rainfall indicate an alkaline pH and a progressive increase in water temperature throughout the day (table 1).

**Table 1:** Physicochemical characteristics of the wastewater from the *Canal San José* during sampling in months of low and high rainfall

Sampling date	Time of sampling (HH:MM:SS)	pH	Water temperature (°C)	Total dissolved solids (mg/L)	Conductivity (μS/m)	BOD_5_ (mg/L)
08/01/2018	13:22:00	7.67	21.1	203	388	No data
11/01/2018	13:41:00	7.69	21.1	259	518	No data
14/01/2018	09:00:00	8.21	16.5	409	776	No data
05/02/2018	09:36:00	8.34	16.7	152	333	No data
07/02/2018	11:12:00	8.54	17.0	305	599	64
09/02/2018	09:05:00	8.33	15.9	513	1,097	91
05/03/2018	09:45:00	8.41	17.2	290	582	102
07/03/2018	12:00:00	8.42	18.0	366	730	243
09/03/2018	13:55:00	7.80	17.7	338	678	173
10/04/2018	11:45:00	8.53	19.3	358	723	3
11/04/2018	07:05:00	8.39	14.5	465	928	180
15/04/2018	06:00:00	8.12	14.7	538	1,078	68
06/05/2018	06:00:00	8.10	15.8	449	898	129
08/05/2018	11:30:00	7.95	19.7	319	637	No data
10/05/2018	13:43:00	8.00	16.9	365	754	No data
05/06/2018	13:30:00	8.10	19.7	331	489	No data
07/06/2018	09:20:00	8.33	15.8	407	502	No data
11/06/2018	09:56:00	8.21	16.1	398	450	No data

BOD_5_: Biochemical oxygen demand over five days

### Quantification of group A rotavirus in water samples

Our results show contamination by group A rotavirus in wastewater from the Canal San José, with concentrations between 7.90 and 79.2 copies/L, exceeding the calculated detection threshold of 7.8 copies/L(table 2).

**Table 2:** Rotavirus concentration in water samples. Copies of group A rotavirus were detected in low and high rainfall months on wastewater samples from the *Canal San José* in *La Ramada*, a rural area in the west of Bogotá. Water samples with quantification cycle (Cq) above 40 were considered negative. Water samples below the detection threshold (7.8 copies/L) were considered negative.

Water sample date	Rainy season		Group A rotavirus	Below detection threshold
	Low-rainfall months	High-rainfall months	Copies/L (mean)	
08/01/2018	X		22.4	
11/01/2018	X		11.3	
14/01/2018	X		9.24	
05/02/2018	X		17.1	
07/02/2018	X		8.57	
09/02/2018	X		0.692	X
05/03/2018	X		8.57	
07/03/2018	X		66.3	
09/03/2018	X		76.5	X
10/04/2018		X	8.57	
11/04/2018		X	7.90	
15/04/2018		X	8.06	
06/05/2018		X	5.00	
08/05/2018		X	9.24	
10/05/2018		X	8.23	
05/06/2018	X	X	2.53	X
07/06/2018	X		1.57	X
11/06/2018	X		0.460	X

### Rotavirus infection of isolated intestinal villi_

**Figure 3:**
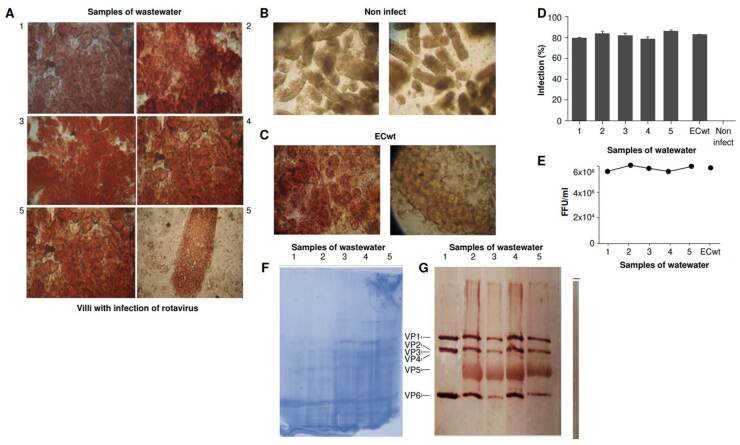
Isolated intestinal villi infected with rotavirus found in wastewater samples from the *Canal San José* in the *La Ramada* network. Intestinal villi isolated from a mouse were suspended in MEM and seeded in 96-well cell culture plates. After 24 hours of incubation at 37 °C, the villi were harvested. **A)** Immunohistochemical analysis using antibodies to detect structural and non-structural rotavirus proteins: VP1-VP6, and NSP4, NSP6. The percentage of infected cells was calculated per triplicate, and each sample was analysed twice; **B)** Negative control: Mock-infected villus cells; **C)** Positive control: Villi infected with wildtype mouse rotavirus strain (ECwt); **D)** Percentage of infected cells in villi converted into focus-forming units (FFU); **E)** Virion production yield expressed as FFU; **F)** SDS-PAGE of the samples used for western blot, staining with Coomassie blue; **G)** Western blot using antibodies against rotavirus structural proteins.

Villi were inoculated with 100 µl of concentrated samples pre-treated with trypsin (10 µg/ml), then incubated at 37 °C for 30 minutes, and harvested 24 hours later. Of the 18 samples collected, 12 tested positive, meaning they successfully infected the villi isolated from mouse intestines. Immunohistochemical images of five representative samples are shown in figure 3A. As a negative control, we added only culture medium (figure 3B), and as a positive control, we infected villi with the wild-type mouse rotavirus ECwt (figure 3C). The infection rate in the villi was approximately 80%, similar to that of the positive control (figure 3D). The virion production yield was around 6 × 10^8^ FFU/ml (figure 3E). Each one of the five representative samples was used to infect new villi to obtain sufficient material for protein analysis by SDS-PAGE, Coomassie blue stain (figure 3F), and Western blotting using antibodies against rotavirus structural proteins (figure 3G).

## Discussion

Our results indicate rotavirus values lower than those reported in other studies on enteric viruses found in raw wastewaters. For example, some studies have reported rotavirus concentrations between 2and 720 copies/ ml ^5^^,^^8^^,^^9^ in wastewaters. Several authors report enterovirus concentrations (poliovirus, coxsackievirus A, coxsackievirus B, echovirus) in raw wastewater of 1.0 × 10^2^ to 2.4 × 10^6^ copies/ml ^6^^,^^22^^-^^24^.

Regarding adenovirus, concentrations between 1.0 and 1 × 10^6^ copies/ ml have been evidenced in this type of water ^6^^,^^25^. Additionally, researchers have reported norovirus concentrations (genogroup I/II) between 4.8 × 10^5^ and 9.3 × 10^6^ copies/ml in raw wastewater ^4^^,^^6^, in contrast with those reported for human astroviruses between 8.1 × 10^2^ and 2.1 × 10^7^ copies/ml ^5^^,^^26^. The low concentrations of group A rotavirus detected in this study may be attributed to inhibitory factors associated with the enzymatic reaction of the qRT-PCR, likely related to water quality conditions. This limitation should be carefully considered and addressed in future studies ^27^.

The late cycle threshold (Ct) confirms a low concentration of viruses. However, infected villi indicate the presence of virions with infective capacity, able to easily replicate in this model under ideal conditions ^17^^,^^21^. Not all group A rotaviruses can grow in intestinal villi. However, strains derived from different organisms -including mouse, human, and pig- have been tested and shown to infect villi in a similar way. These results highlight the utility of the isolated villi model for supporting the replication of wild-type rotaviruses ^21^. In this regard, this model is better than other cell lines, like MA-104 or Vero, commonly used in viral infection assays.

This work demonstrates that villi from mouse intestines provides a reliable *ex vivo* model for isolating wild-type rotavirus from wastewater. These findings suggest that these villi express the necessary receptors used by human rotaviruses to infect the human gut. These results align with the group A rotavirus detection through qRT-PCR.

Additionally, this study demonstrates that both techniques are complementary. While qRT-PCR is highly sensitive and specific for detecting rotavirus genomes, it cannot distinguish between infectious and non- infectious particles. However, through the villi mouse model, we provide an alternative approach to determine whether rotaviruses identified by molecular techniques are indeed infectious. These results are similar to those reported by other studies, like the one of Jordan *et al*., which identified human norovirus in wastewater using molecular methods (qRT-PCR) and determined its viability using a model of mouse intestinal villi ^17^.

Based on our data, rotavirus concentrations in wastewater are a reliable proxy to monitor the environmental health of these waters in Colombia, using a molecular epidemiologic approach. These results are consistent with a public health surveillance perspective, including wastewater quality ^12^ for detecting rotavirus, other enteric viruses, and coronaviruses such as SARS- CoV-2 ^1^^,^^3^^,^^11^. Other authors have evaluated the molecular epidemiology of human noroviruses in other waters -drinking water ^10^^,^^28^, stormwater, leachates, and river water ^10^- as well as human enteroviruses and adenoviruses in river water ^20^.

Regarding the periods of high and low rainfall, our results showed the highest concentrations of human rotavirus in wastewater during the period of low rainfall (January, February, and March). This finding coincides with a study carried out for this type of water in Caracas, Venezuela, showing higher prevalence of human rotavirus in February, March, and September ^29^. Another study conducted in Brazil determined a strong association between the dry season and the presence of rotavirus A ^30^.

It is important to consider that the wastewater analyzed in our study is reused for the irrigation of fresh vegetables, which are later consumed in Bogotá and the surrounding areas ^10^. Therefore, it is imperative to create public policies for the epidemiological surveillance of human enteric pathogens in these waters ^1^^,^^11^^,^^13^^,^^14^ and to lay the groundwork for the management of basic sanitation of irrigation systems ^13^^,^^14^. Upon reviewing the literature on the concentration of human enteric viruses in wastewater, we found that such data can be modelled ^1^, but our dataset was insufficient.

Finally, it is worth mentioning that, a few years ago, levels of human norovirus infection in mouse intestinal villi had already been reported in drinking water samples from a school in the Usme district in Bogotá ^17^. Together with our findings -regarding the infective capacity of human rotaviruses in irrigation water- these results highlight the need to reorient research and public policies on water quality surveillance in Colombia. These policies should include water for human consumption and must incorporate infectivity assays and molecular tests to better interpret, analyse, and evaluate the risks for human, animal, and environmental health. In addition to the above and given the lack of practical access to these methodologies in the country, it is crucial to establish at least one reference research laboratory, from which this level of knowledge and development can be further advanced and replicated.
